# Sustainable Production of Ginsenosides: Advances in Biosynthesis and Metabolic Engineering

**DOI:** 10.3390/plants14182821

**Published:** 2025-09-09

**Authors:** Yang Xue, Ruixiang Zhang, Tie Li, Qindi Deng, Weidong Luo, Ruyue Chang, Dongchang Zeng, Jiantao Tan, Tianhu Sun, Yao-Guang Liu, Yang Xiang, Qinlong Zhu, Nan Chai

**Affiliations:** 1 Guangdong Basic Research Center of Excellence for Precise Breeding of Future Crops, Guangdong Laboratory for Lingnan Modern Agriculture, State Key Laboratory for Conservation and Utilization of Subtropical Agro-Bioresources, College of Agriculture, South China Agricultural University, Guangzhou 510642, China; 2 University Engineering Research Center of Bioinformation and Genetic Improvement of Specialty Crops, Guangxi, College of Life Sciences, Guangxi Normal University, Guilin 541006, China; 3 Rice Research Institute, Guangdong Academy of Agricultural Sciences, Guangzhou 510640, China; 4 Department of Biological Sciences, East Tennessee State University, Johnson City, TN 37614, USA; 5 Guizhou Institute of Crop Germplasm Resources, Guizhou Academy of Agricultural Sciences, Guiyang 550006, China

**Keywords:** ginsenoside biosynthesis, synthetic biology, sustainable production, plant chassis, ginsenosides, natural products

## Abstract

Ginsenosides, the primary bioactive components of *Panax ginseng*, exhibit diverse pharmacological properties, ranging from anticancer to neuroprotective effects. However, traditional production by *ginseng* cultivation faces limitations due to extended growth cycles, insufficient yields, intricate extraction processes, and significant environmental dependencies. Synthetic biology and synthetic metabolic engineering offer promising alternatives for sustainable manufacturing of essential bioactive compounds, including ginsenosides. First, this review describes the ginsenoside biosynthesis pathways, emphasizing crucial enzymes (e.g., HMG-CoA reductase, squalene epoxidase, dammarenediol-II synthase, amyrin synthase, and various UDP-glycosyltransferases) and their regulatory networks. Understanding these fundamental pathways enables rational engineering of production systems. Second, it examines current synthetic biology approaches, encompassing plant cell, tissue, and hairy root cultures, engineered microbial hosts including *Saccharomyces cerevisiae* and *Escherichia coli*, and cell-free enzymatic synthesis. Third, it evaluates the medicinal significance, market prospects, and industrial feasibility of these biomanufactured compounds. Finally, it analyzes the sustainability of production models and explores the emerging potential of engineered plant chassis. These advanced methodologies directly address traditional agricultural constraints and establish a robust framework for future ginsenoside synthesis.

## 1. Introduction

Asian *ginseng* (*Panax ginseng* C.A. Meyer; hereafter “*ginseng*”) belonging to the Araliaceae family is a perennial and highly valued medicinal plant, known as “the king of medicinal herbs” [1]. *Panax* L. is cultivated in 35 countries worldwide, with Asia being the primary production region [1,2]. China possesses extensive *ginseng* resources and is the leading producer of *ginseng*, contributing approximately 70% of the global output. The principal bioactive constituents of *Panax* L. are triterpenoid ginsenosides, a class of glycosylated triterpene compounds and the most sought-after *ginseng* secondary metabolites. Ginsenosides have a medicinal tradition spanning more than 2000 years [3,4], exhibiting significant neuroprotective effects and pharmacological activities, including anticancer, antioxidant, anti-aging, anti-inflammatory, and anti-apoptotic activities [5]. Ginsenosides Rg_1_ and Rb_1_ are the most abundant constituents, each accounting for more than 20% of the total saponin content. Furthermore, Panax notoginseng possesses several unique saponins, such as notoginsenosides R_1_, Rt, R_2_, R_3_, R_4_, and R_6_ [6]. Notoginsenoside R_1_ is the third most abundant saponin in Panax notoginseng [7]. In contrast, Panax japonicus is characterized by a notably high content of oleanane-type saponins [8]. Approximately 180 structurally distinct ginsenosides have been characterized and isolated from 17 *Panax* species [9,10,11].

Traditional *ginseng* field cultivation faces several challenges. As a slow-maturing perennial crop, *ginseng* requires 6–7 years of growth before reaching harvestable maturity. Its strict agroecological requirements, combined with replanting issues, lead to soil depletion and increased susceptibility to pathogens and pests [12,13]. As *ginseng* cultivation expands, insufficient production management techniques and inappropriate pesticide application considerably affect both yield and quality of *ginseng*. Notably, the saponin content of *ginseng* remains low, with total ginsenosides constituting merely 2–3% of dry weight, while highly bioactive rare ginsenosides occur at concentrations below 0.01% [11]. Traditional agricultural production cannot meet market demands. The complex structure of triterpenoid saponins presents considerable challenges for direct extraction and purification, further complicated by hazardous organic solvent waste generation. Although chemical synthesis could theoretically provide an alternative production method, the stereochemical complexity of ginsenoside molecules renders synthetic approaches impractical in terms of technical feasibility and cost-effectiveness [14].

Synthetic biology presents opportunities for sustainable ginsenoside production. Current research priorities include metabolic pathway engineering to increase target metabolite yields and synthetic biology-based production platforms. Advances in ginsenoside pathway elucidation, transcriptional network regulation, and microbial chassis development have established essential knowledge for biosynthesizing ginsenosides and their analogs by metabolic engineering, enabling yield optimization by artificial pathway control in heterologous hosts. This review comprehensively documents state-of-the-art technologies and innovative approaches in ginsenoside biosynthesis research. It examines recent progress in ginsenoside biosynthesis pathways, critical enzyme genes, transcriptional regulation, and microbial cell engineering approaches, while outlining future development trends for subsequent investigations in relevant fields.

## 2. The Biosynthetic Pathway of Ginseno Sides

### 2.1. Classification of Ginsenosides

**Figure 1:**
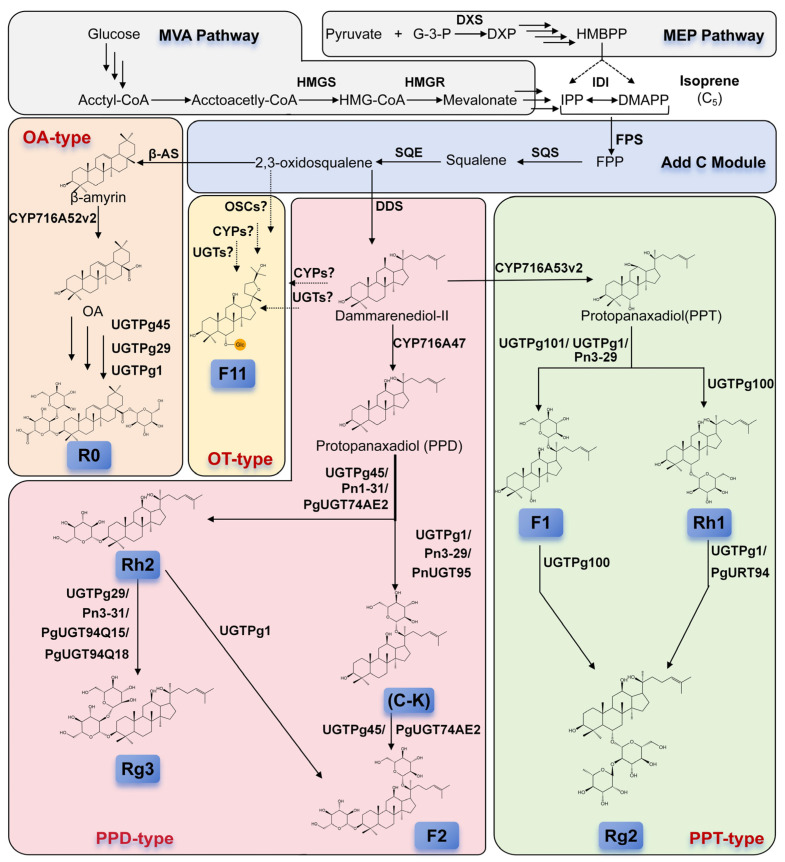
Biosynthetic pathway of ginsenosides. G-3-P, Glyceraldehyde 3-phosphate; DXP, 1-Deoxy-D-xylulose 5-phosphate; HMBPP, 4-Hydroxy-3-methylbut-2-enyl diphosphate; HMG-CoA, 3-Hydroxy-3-methylglutaryl-coenzyme A; IPP, isopentanyl pyrophosphate; DMAPP, 3,3-dimethylallyl diphosphate; FPP, farnesyl pyrophosphate; FPS, farnesyl pyrophosphate synthase; SQS, squalene synthase; SQE, squalene epoxidase; DDS, dammarenediol synthase; CYP716A47, CYP716A53v2, and CYP716A52v2 are all Cytochrome P450 oxidases enzymes; PPD, protopanaxadiol; PPT, protopanaxatriol; β-AS, β-amyrin synthase; OA, oleanolic acid. R0, F11, Rh2, Rg3, C-K, F2, F1, Rh1, and Rg2 are all commonly used abbreviations for various ginsenosides. The background colors indicate different biosynthetic pathways: gray for the precursor synthesis MEP and MVA pathways; blue for the carbon chain elongation process; orange for the biosynthesis of OA-type ginsenosides; light yellow for OT-type; pink for PPD-type; and green for PPT-type ginsenosides.

Ginsenosides are glycosidic natural compounds consisting of a triterpenoid saponin skeleton associated with one or more glycosyl groups. The triterpenoid saponin skeleton is composed of 30 carbon atoms. Based on differences in their aglycone skeleton and the position and number of glycosyl groups, ginsenosides are commonly classified into (1) dammarane-type tetracyclic triterpenoid saponins, including protopanaxadiol (PPD)-type (comprising prototypical members such as compound K [CK] and Rg1, where glycosylation occurs at β-hydroxyl [OH] groups of C3 and/or C20) and protopanaxatriol (PPT)-type (comprising prototypical members such as Rb1 and Rh1 [15], where glycosylation occurs at α-OH groups of C6 and/or β-OH groups of C6 and/or C20); (2) oleanane (OA)-type pentacyclic triterpenoid saponins derived from oleanolic acid aglycones [16,17]; and (3) ocotillol (OT)-type tetracyclic triterpenoid saponins, and this glycosyl groups are attached at C20 and C24 positions, forming four possible configurations: (20S, 24S), (20R, 24R), (20S, 24R), and (20R, 24S) (Figure 1). Representative compounds include F11, RT5, and RT2 [18,19,20]. *Panax* L. contains PPD-type saponins at 45%–60% [21], PPT-type saponins at 12%–20%, and OA-type saponins at 7%–10% [5]. For ocotillol-type saponins, which are derivatives of the dammarane family, serve as characteristic and marker constituents of American ginseng (*Panax quinquefolius* L.). Nevertheless, their content remains relatively low, with a representative compound, pseudoginsenoside F11, typically occurring at a level of approximately 0.1% [22,23]. In ginsenoside nomenclature, the prefix “R” indicates “Root”, reflecting the accumulation of ginsenosides primarily in *ginseng* roots, while the suffix “x” denotes the chromatographic elution order based on alphabetical polarity ranking [24]. Naturally occurring ginsenosides exhibited poor absorption in their intact form with short retention times in in vivo animal studies, resulting in low biological utilization rates (typically below 10%) [25,26]. However, deglycosylation of ginsenosides substantially improves their absorption rate [27]. Due to their limited natural abundance, ginsenosides obtained by deglycosylation of more common ginsenosides are termed rare ginsenosides. These rare ginsenosides demonstrate enhanced bioavailability [28].

### 2.2. Biosynthetic Pathway of Ginsenosides

Recent advances in multi-omics research related to *ginseng* have clarified the biosynthetic pathways of ginsenosides. These pathways encompass multiple enzymatic reactions, consisting of up to 20 distinct steps [29] (Figure 1). Starting with isoprene as the precursor, ginsenoside molecules are synthesized through a sequence of skeletal formation, modification, and glycosylation reactions, resulting in structurally diverse compounds. However, current research on OT-type (ocotillol-type) ginsenosides remains relatively limited. These compounds are characteristic constituents primarily identified in the stems, leaves, and roots of American ginseng (*Panax quinquefolium*). Ocotillol-type saponins belong to a class of tetracyclic triterpenes featuring a furan ring at the C20 position [22]. It is hypothesized that their biosynthetic backbone may originate from protopanaxadiol (PPD), followed by modifications mediated by cytochrome P450 enzymes and glycosyltransferases [23].

#### 2.2.1. Upstream Biosynthesis Pathway of Isoprenoid Precursors

The process begins with Acetyl-CoA and concludes with the formation of the terpenoid biosynthesis precursors isopentenyl diphosphate (IPP) and isomer dimethylallyl diphosphate (DMAPP). The mevalonate (MVA) pathway commences with Acetyl-CoA, advancing through HMG-CoA synthesis, followed by reduction to mevalonic acid catalyzed by the rate-limiting enzyme HMG-CoA reductase (HMGR).

In *Saccharomyces cerevisiae*, *HMGR* overexpression significantly increased the downstream isoprenoid flux [30]. In addition, *ginseng* contains multiple HMGR isogenes displaying distinct spatiotemporal expression patterns. *Ginseng* has two functional copies: *PgHMGR1* likely supports secondary metabolite biosynthesis, whereas *PgHMGR2* putatively contributes to root-specific ginsenoside accumulation [31]. In *Panax* roots, the MVA pathway primarily contributes to ginsenoside biosynthesis [32]. The methylerythritol 4-phosphate (MEP) pathway begins with pyruvate and glyceraldehyde 3-phosphate, with 1-deoxy-D-xylulose-5-phosphate (DXP) synthase and DXP reductoisomerase acting as key catalytic enzymes. DXP synthase overexpression leads to an increased production of downstream terpenoid biosynthetic products [33,34]. Coordination between MVA and MEP pathways makes sufficient precursors available for successful downstream metabolic engineering strategies.

#### 2.2.2. Formation of the Triterpenoid Saponin Skeleton: Squalene and Its Cyclization

Farnesyl diphosphate synthase (FPS) catalyzes the sequential condensation of IPP with DMAPP to form geranyl diphosphate (GPP, C_10_), followed by IPP addition to GPP producing farnesyl diphosphate (FPP, C_15_). The mechanism involves the nucleophilic attack of IPP on the electrophilic allylic diphosphate (DMAPP/GPP). *PgFPS* participates in the biosynthesis of ginsenosides and phytosterols, functioning as a crucial enzyme for ginsenoside formation [35]. Squalene acts as a vital biosynthetic precursor for triterpenoids and their derivatives, with its production regulated by squalene synthase (SQS) [36]. In *ginseng*, all three *PgSQS* genes encode enzymes, but *PgSQS2* and *PgSQS3* exhibit tissue-specific expression patterns detectable only in particular organs [37]. The final step, catalyzed by squalene epoxidase (SQE), produces 2,3-oxidosqualene [38,39]. In addition, both PgSQE1 and PgSQE2 from *ginseng* leaves and adventitious roots were cloned, and these two genes exhibited distinct regulatory mechanisms, with *PgSQE1* being implicated in ginsenoside biosynthesis [39].

2,3-Oxidosqualene represents a critical metabolic branch point, cyclized by specific oxidosqualene cyclases (OSCs). The *ginseng* genome contains 19 distinct OSC genes [40]. Among these, dammarenediol-II (DD) synthase (DDS) catalyzes the cyclization of 2,3-oxidosqualene to synthesize DD. DDS gene overexpression enhances the accumulation of dammarane-type ginsenosides in *Panax* species, establishing DDS as a key rate-limiting enzyme in this biosynthetic pathway [41]. β-Amyrin synthase (β-AS) catalyzes the cyclization to produce β-amyrin. This enzyme remains the only enzyme identified that synthesizes OA-type ginsenoside Ro. These cyclization steps determine the fundamental carbon skeleton structures of different ginsenoside classes [42].

#### 2.2.3. Key Modifications Mediated by Cytochrome P450 (CYP450) Enzymes

Plant CYP450 enzymes constitute a supergene family comprising multiple subfamily members. These heme-containing oxidases, encoded by CYP450 genes, contribute to diverse biological processes, including the post-modification steps of triterpenoid saponin secondary metabolism. In ginsenoside biosynthesis, CYP450 enzymes primarily facilitate complex modifications, such as hydroxylation, oxidation, and glycosylation of the carbon skeleton. These intricate CYP450-mediated modifications are primarily responsible for generating structural diversity among ginsenosides. Several key enzymes have been identified. The CYP716A47 gene encodes an enzyme that catalyzes the C12 hydroxylation of DD, converting it to PPD [43]. The gene product of CYP716A53v2 functions as a hydroxylase catalyzing the conversion of PPD to PPT, representing a critical step in the formation of PPT-type triterpenoid aglycones during ginsenoside biosynthesis [44,45,46]. Furthermore, CYP716A52v2 catalyzes the synthesis of OA-type ginsenosides from β-amyrin [47].

#### 2.2.4. Pivotal Role of UDP-Glycosyltransferases (UGTs) in Generating Structural Diversity

UGTs catalyze the glycosyltransferases reaction, representing the final critical step in ginsenoside biosynthesis [48]. This process transfers a glycosyl moiety from an activated sugar donor to the aglycone scaffold of ginsenosides, thereby modulating key properties such as bioactivity, solubility, and stability [49]. Notably, the variation in aglycone structures combined with differences in the number and position of attached glycosyl moieties confers distinct biological functions upon individual ginsenosides, explaining the remarkable structural and functional diversity observed within this class of compounds [50,51]. In plants, UGTs belong to a highly specific gene family, with different UGTs specific to both glycosyl donors and glycosyl acceptors. In addition, UGTs demonstrate regioselectivity, selectively acting on the same position of one or more acceptor substrates with similar structures [52,53].

**Figure 2:**
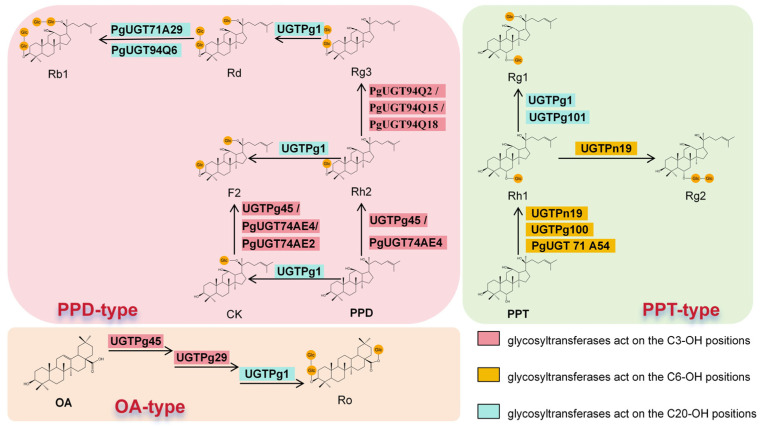
Glycosyltransferases: Types and Action Sites.

The currently identified glycosyltransferases primarily act on the dammarane- and oleanane-type, mainly adding glucosyl moieties at the C3-OH, C6-OH, and C20-OH positions (Figure 2). For the PPD skeleton, Glycosyltransferases can also directly transfer the glucosyl moiety to the C20-OH group of PPD, like UGTPg1, etc. UGTPg1 exemplifies this activity, as it modifies the C20-OH to synthesize ginsenoside compound K [30], also can catalyze the conversion of ginsenosides Rg3 and Rh2 to Rd and F2 [30], respectively. Furthermore, PgUGT71A29 or PgUGT94Q6 can further glycosylate Rd to synthesize ginsenoside Rb1 [54,55]. Similarly, glycosylation at the C3-OH of PPD is facilitated by specific enzymes like UGTPg45. it (or PgUGT74AE4) can synthesize ginsenoside Rh2 [56,57]; both UGTPg45 (PgUGT74AE4) and PgUGT74AE2 can synthesize ginsenoside F2 using ginsenoside CK as a substrate [56,57]. Based on this, PgUGT94Q2, PgUGT94Q15, and PgUGT94Q18 are involved in the synthesis of ginsenoside Rg3 [58]. For the PPT skeleton, glycosyltransferases are utilized to synthesize corresponding ginsenosides by glycosylating the hydroxyl groups at the C6-OH and C20-OH positions. UGTPn19 or UGTPg100 (PgUGT71A54) can glycosylate and modify the C6 hydroxyl group to produce the rare ginsenoside Rh1 [59,60]. Additionally, UGTPg101, UGTPg1, and UGTPn19 can specifically catalyze the glycosylation of both the C20-OH and C6-OH of PPT, enabling the biosynthesis of Rh1 and F1 [60]. Furthermore, Rg2 can be synthesized through catalysis by UGTPn19 [61]. In the case of the oleanane-type skeleton, the rare ginsenoside Ro is ultimately synthesized via the individual actions of UGTPg45, UGTPg29, and UGTPg1 [62]. Therefore, UGTs play a crucial role in the biosynthesis of ginsenosides.

## 3. A Paradigm Shift for Sustainable Ginsenoside Production

**Figure 3:**
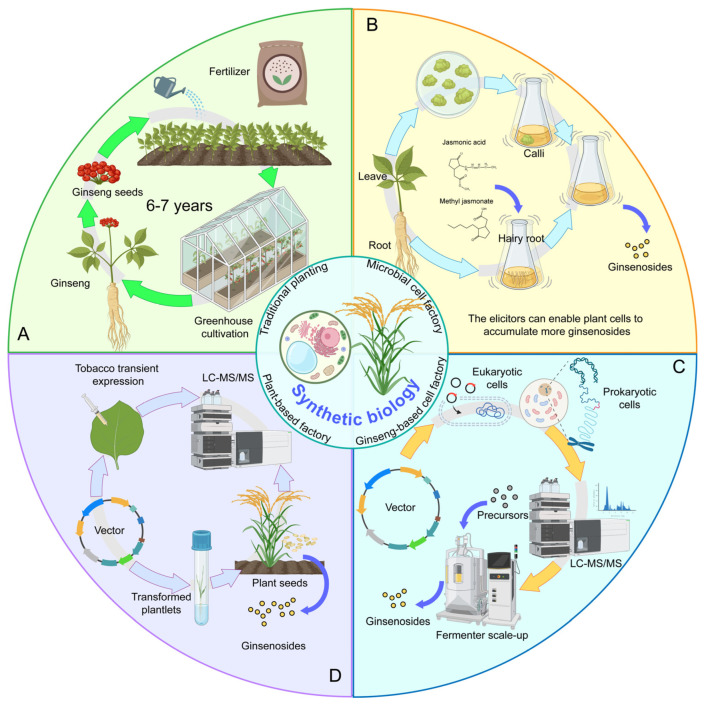
Sustainable production of ginsenosides driven by synthetic biology. (**A**): Innovation in *Panax* L. cultivation techniques. (**B**): Development of tissue culture techniques based on *Panax* L., including cells, tissues, and hairy Roots. (**C**): Microbial chassis-based biosynthesis of ginsenosides, including biosynthesis by microorganisms and enzymatic biocatalysis synthesis. (**D**): Plant chassis-based new models for ginsenoside production.

Traditionally, ginsenosides have been sourced from the cultivated *Panax ginseng* plant. However, their production faces challenges such as the long growth cycle of ginseng and its stringent environmental requirements. While further improvements in cultivation techniques remain important, the advancement of synthetic biology and genetic engineering has opened up novel strategies for ginsenoside synthesis and production. These approaches include ginseng tissue culture, the construction of microbial cell factories, and the heterologous production of ginsenosides in other plant systems. These innovations have breathed new life into the biotechnological synthesis of ginsenosides (Figure 3).

### 3.1. Innovations in Agricultural Technology

The consumption of ginseng has a history of over 1000 years, yet its cultivation remains challenging. With the advancement of modern agriculture, ginseng cultivation techniques have undergone significant innovations (Figure 3A): selecting superior wild ginseng strains, artificially nurturing seedlings, and subsequently transplanting them for cultivation. Due to its preference for cold climates and sensitivity to heat, ginseng requires specific environmental temperature conditions. Therefore, shaded canopy structures are often erected to facilitate germination and growth. Fertilization is also essential in ginseng cultivation, with an emphasis on the use of humic acid, organic fertilizers, and microbial-based fertilizers. Additionally, preventing pests and diseases during growth is critical, and the application of natural botanical pesticides or low-toxicity chemical pesticides is recommended. Furthermore, recent studies have shown that the amount of major protopanaxadiol-type ginsenosides increased under shade and red light conditions [63]. While innovations in agricultural technology can enhance the yield of ginsenosides, market demand remains difficult to meet.

### 3.2. Plant-Based Systems: Ginseng Cell, Tissue, and Hairy Root Cultures

*Ginseng* plant tissues and organs, including roots, stems, leaves, flowers, and fruits, synthesize ginsenosides, with content and types varying among different organs [64,65]. For instance, in roots or hairy roots, ginsenoside Rb1 concentration is high. During leaf sprouting from the previous year’s roots [66], the ginsenoside content significantly increases in leaves while decreases in roots, demonstrating that ginsenosides undergo dynamic synthesis during annual growth [4].

Cell and tissue culture methods have been extensively used to mitigate the vulnerability of field cultivation to environmental factors. *Ginseng* cell suspension culture represents an effective approach for ginsenoside production (Figure 3B). In *ginseng* cell culture, Murashige and Skoog medium supplemented with plant growth regulators, such as 2,4-dichlorophenoxyacetic acid and naphthalene acetic acid, is commonly utilized [67]. In addition, the incorporation of sugar molecules into the medium significantly enhances ginsenoside yield. Compared with media of 30 g·L^−1^ sucrose concentration, media of 60 g·L^−1^ sucrose can increase the ginsenoside content by 3.5-fold [68]. Similarly, sorbitol addition results in a 3.5-fold increase in ginsenoside yield compared with control containing casein hydrolysate and 30 g·L^−1^ sucrose [69].

Adventitious root culture demonstrates high ginsenoside production capacity and maintains stability under physical and chemical conditions during large-scale cultivation, making it suitable for industrial-scale ginsenoside production [70]. Adventitious roots derived from calli of petioles, lateral roots, and floral bud primordia of *ginseng* and *P. notoginseng* (*notoginseng*) have been used to extensively produce ginsenosides [67,71,72].

Hairy roots are transgenic root systems generated by inducing *ginseng* explants with *Agrobacterium rhizogenes* [73]. These roots develop through plant transformation by Ri plasmid T-DNA (mediated by *Agrobacterium*), where four “rol” genes (*rolA*, *rolB*, *rolC*, and *rolD*) in T-DNA are essential for hairy root formation. They stimulate root development by modulating phytohormone balance, and auxin and cytokinin metabolism. The rolB gene serves as the primary regulator of secondary metabolism, capable of activating specific transcription factors for most metabolic pathways [74]. *Ginseng* hairy roots exhibit advantages including rapid growth rate, hormone autotrophy, stable genetic properties, abundant secondary metabolites, and high saponin content. Advancing beyond traditional shaking flask culture, a bioreactor designed for hairy roots has been developed. It provides gentle mixing and oxygenation by wave-like agitation, preventing mechanical damage typically caused by conventional stirring. By optimizing culture conditions (medium replacement every 14 days), the initial fresh root weight increased by over 28-fold, while saponin production showed nearly 3-fold improvement compared with shaking flask culture [75]. In hairy root culture, microbubble generators enhance oxygen transfer efficiency while minimizing root system shear damage. When combined with surfactants such as Triton X-100, secondary metabolite secretion and yield increase further [76].

Elicitors enable enhanced ginsenoside accumulation in plant cells. Methyl jasmonic acid (MeJA) represents the most effective elicitor [70,77], increasing the gene expression levels of HMGR, SQS, SQE, and DDS, thereby enhancing ginsenoside content [36,77,78,79]. After 7 days of MeJA treatment, PPD-type ginsenosides increased by 5.5- to 9.7-fold, while PPT-type saponins showed 1.9- to 3.8-fold increase. In *notoginseng*, the addition of 200 μmol·L^−1^ MeJA results in differential distribution of monomeric saponin types and content, with PPD-type ginsenosides increasing 9-fold and PPT-type ginsenosides increasing 2-fold [80]. Jasmonic acid (JA) significantly influences PPD-type ginsenoside accumulation [81]. Moreover, the addition of JA and MeJA to *ginseng* suspension cultures or their application to adventitious and hairy roots enhances ginsenoside content. Cell suspension cultures of *ginseng* and *notoginseng* treated with 200 μmol·L^−1^ MeJA exhibited significant elevation in PPD-type ginsenosides [82].

### 3.3. Microbial Synthesis of Ginsenosides

Microbial cell factories present notable advantages, including rapid growth, established genetic tools, compatibility with scalable fermentation, and renewable feedstock utilization, making them optimal for producing plant secondary metabolites such as ginsenosides [48] (Figure 3C). This marks a significant transition from plant-based systems toward improved efficiency and scalability. The primary microbial chassis used for ginsenoside synthesis include prokaryotic cells (*Escherichia coli* [*E. coli*] and *Bacillus subtilis* [*B. subtilis*]) and eukaryotic cells (*Yarrowia lipolytica* and *S. cerevisiae*). *E. coli*, due to its rapid growth, well-defined genetic background, and straightforward genetic manipulation, has emerged as the preferred chassis cell for natural product synthesis. For instance, the engineered reconstruction of exogenous MVA and farnesene biosynthetic pathways in *E. coli* achieved efficient production of farnesene at 1.1 g/L [83]. The *B. subtilis* host cell is generally recognized as safe and classified as food-grade; it provides substantial advantages including inherent safety (endotoxin-free), simple fermentation requirements, high cell density growth, and robust protein secretion capacity. For example, by optimizing culture temperature and metabolic pathways, squalene production reached 7.5 mg/L with *B. subtilis* as the chassis [84]. Compared with prokaryotic cells, eukaryotic cells demonstrate distinct advantages in heterologous biosynthesis of ginsenosides. The endogenous MVA pathway enables consistent supply of the ginsenoside precursors IPP, DMAPP, and squalene, while the abundant intracellular membrane structures and post-translational modification systems facilitate the catalytic functions of cyclases and CYP450 oxidases. For instance, heterologous expression of key MVA pathway genes coupled with NADPH-CYP450 monooxygenase in yeast cells achieved production of 161.8 mg/L ginsenoside CK [85].

For microbial systems, the chassis cell determines the biosynthetic yield of ginsenosides and their derivatives. Using synthetic biology approaches, directed chassis engineering optimizes endogenous metabolic flux and energy utilization efficiency in these cells, thus enhancing target compound production. With advancements in synthetic biology applications for microorganisms, multiple ginsenosides have been successfully synthesized in microbial systems (Table 1).

### 3.4. Enzymatic Biocatalysis for Ginsenoside Production

Enzymatic biotransformation uses purified or recombinant enzymes (ginsenosidases, specific UGTs, CYP450s) to catalyze defined reaction steps in vitro, converting abundant major ginsenosides into high-value rare ginsenosides. This methodology presents several advantages: rapid reaction cycles, reduced contamination risk, enhanced product purity, precise controllability, and specific substrate selectivity [11] (Figure 3B). Enzymes with distinct characteristics selectively cleave glycosidic bonds of specific configurations and compositions, facilitating the directed synthesis of target compounds. Common microbial sources include bacterial genera *Flavobacterium*, *Burkholderia*, *Microbacterium*, and *Agrobacterium*, alongside fungi such as *Aspergillus* spp., *Penicillium* sp., and *Emericella vermicola* [97].

The enzymatic hydrolysis of the PPT-type ginsenoside mixtures was performed using crude enzyme preparations from galactosidase (*Aspergillus* spp.) and lactase (*Penicillium* sp.), producing significant quantities of ginsenosides Rg2 and Rh1 [98]. Lactase and β-galactosidase (*Aspergillus* spp.) effectively convert PPD-type saponin mixture into ginsenosides F2 and CK, cellulase (*Trichoderma viride*) facilitates their transformation into ginsenoside Rd, and lactase (*Penicillium* sp.) catalyzes the conversion of PPD-type saponin mixture into ginsenosides Rd, Rg3, and CK [99]. In addition, the recombinant β-glycosidase from *Sulfolobus solfataricus*, expressed in *E. coli*, transformed *ginseng* root extract to ginsenoside CK with a conversion yield of 80.5% in an intracellular reaction [100]. Similarly, the recombinant β-glycosidase from *Pyrococcus furiosus* expressed in *E. coli* converted *ginseng* root extract primarily into ginsenoside CK with a conversion yield of 79.5%, and transformed PPD-type ginsenoside with 100% efficiency, achieving a final PPD-type ginsenoside yield of 1.8 g·L^−1^ [101].

## 4. Ginsenoside Bioprospecting: Advances and Applications

### 4.1. Pharmacological Significance and Multifaceted Bioactivities

Ginsenosides exhibit a broad spectrum of pharmacological activities, including anticancer, anti-inflammatory, antioxidant, anti-aging, anti-fatigue [102,103,104,105,106], neuroprotective, immunomodulatory, anti-diabetic, and cardiovascular protective properties. These compounds demonstrate remarkable clinical potential and therapeutic value [107] (Table 2). Their therapeutic capabilities have significantly driven research and development efforts in ginsenoside production methodologies.

The biological activity of ginsenosides depends on their specific structures, which vary according to the type of aglycone (e.g., PPD, PPT, OA), the number of sugar groups, and the sites of glycosylation. The anticancer properties of ginsenosides, particularly those of rare ginsenosides such as Rg3, Rg5, and CK, are among the most extensively studied areas (Table 2). Their effects are multi-target in nature. The anticancer mechanisms of ginsenosides can be categorized as follows: induction of apoptosis (CK); cell cycle arrest (Rg3, Rg5, Rk3); and inhibition of metastasis (Rk1). Additionally, one of the most remarkable features of ginsenosides is their bidirectional immunomodulatory effects (CK, Rh2, Rs6, Rk1, Rk3), which exemplify the concept of an “adaptogen.” Similarly, in terms of antioxidative stress (CK, F1, Rk1), they can scavenge free radicals and enhance the activity of endogenous antioxidant enzymes, such as superoxide dismutase (SOD) and glutathione peroxidase (GSH-Px), thereby protecting neurons from oxidative damage. The above demonstrates that the pharmacological significance of ginsenosides lies in their pleiotropy, they are capable of influencing a broad network of targets rather than a single receptor.

### 4.2. Industrialization Prospects and Market Analysis

Global demand for ginsenosides and *ginseng* extracts remains substantial and exhibits continuous growth. A significant shift is occurring from common to rare/minor ginsenosides. Sales of rare ginsenoside products increased from $406 million in 2017 to $739 million in 2022, demonstrating a compound annual growth rate (CAGR) of 12.7%. This market is expected to expand further at an elevated CAGR of 16.1%, reaching $1.561 billion by 2027 [137]. The *ginseng* extract market, valued at $24.5 million in 2019, is anticipated to maintain consistent growth. Projections indicate that the total ginsenoside market may achieve a multitrillion-dollar valuation by 2050 [48]. Statistical data reveals increasing consumer preference for rare ginsenoside products, indicating their substantial market potential. This surge in demand stems primarily from enhanced consumer acceptance of herbal medicine, increased disposable incomes, and greater awareness of *ginseng*’s health benefits.

Current ginsenoside production capacity remains insufficient to meet market demand, creating an unsustainable situation. Synthetic biology presents a viable alternative for sustainable and scalable production of ginsenosides and their derivatives. This approach shows considerable commercial potential in ginsenoside manufacturing. The global synthetic biology market is predicted to grow at a CAGR of 22.5%, potentially surpassing $20 billion by 2025. China, representing the world’s largest functional food market for ginsenosides, is accustomed to the steadily increasing demand for high-quality saponins. Market analysis indicates that rare ginsenosides (e.g., CK, Rg3, Rh2, etc., which enhanced bioavailability and potent pharmacological effects, and are common targets for biotechnological production), characterized by superior bioavailability and enhanced bioactivity compared with prototype ginsenosides, have achieved significant developmental progress in pharmaceuticals, nutraceuticals, and cosmetics, suggesting expanding commercialization opportunities.

While synthetic biology production of ginsenosides may offer lower costs than plant extraction, with yeast-based production costs of $0.5–25/mg compared with $25–57/mg for plant-derived equivalents, fermentation expenses including raw materials, energy, and downstream processing must remain competitive [48,86,94,138]. Economic viability depends on market demand and pricing for specific target ginsenosides. Widespread adoption of these technologies will be determined by economic factors. Commercial success in ginsenoside synthetic biology requires more than overcoming technological barriers; economic feasibility, complex regulatory pathways, and public acceptance represent equally crucial and interconnected factors.

## 5. Challenges and Prospects

As the primary bioactive components in *ginseng*, ginsenosides demonstrate exceptional value in pharmaceutical and nutraceutical applications due to their complex chemical structures and broad-spectrum biological activities. However, traditional field cultivation and extraction methods encounter significant challenges regarding yield, efficiency, environmental impact, and sustainability, failing to meet the growing global demand for high-quality, structurally specific ginsenosides, particularly rare saponins.

### 5.1. Current Technical Challenges

#### 5.1.1. Insufficient Research on Enzyme Activity and Structural Optimization

While the complete metabolic pathways for ginsenoside biosynthesis have been elucidated, critical natural enzymes in these pathways rarely undergo structural optimization, resulting in low catalytic efficiency. This limitation is particularly evident for ginseng-specific enzymes, where the expression of plant-derived CYP450 enzymes in microbial systems remains a significant bottleneck. These enzymes typically exhibit low activity in heterologous hosts due to factors including inherently low catalytic efficiency, poor solubility, misfolding tendencies, requirements for specific membrane environments, and dependency on specialized electron transport systems. Also, as enzymes catalyzing the terminal glycosylation step in ginsenoside biosynthesis, UGTs are pivotal for generating structural diversity among ginsenosides. Enhanced screening and catalytic efficiency improvements of UGTs would significantly facilitate the biosynthesis of multiple ginsenoside variants.

#### 5.1.2. Pathway Balancing and Metabolic Burden

The biosynthesis of ginsenosides involves an extended metabolic pathway, initiating from central carbon metabolism (e.g., acetyl-CoA) and proceeding through either the MVA pathway or the MEP pathway to produce IPP and DMAPP. These intermediates are subsequently converted into the triterpene backbone (2,3-oxidosqualene), which then undergoes extensive modification by a series of cytochrome P450 oxidases (CYPs) and UDP-glycosyltransferases (UGTs). In most organisms, the pathway from acetyl-CoA to 2,3-oxidosqualene is inherently present. However, strongly diverting metabolic flux toward saponin biosynthesis competes intensely with host growth, potentially leading to reduced ginsenoside production. Intermediates in this pathway serve as common precursors for a variety of essential metabolites; their substantial depletion may result in defective cellular membranes, growth arrest, or even cell death. Moreover, precise coordination of multiple enzymatic steps, such as those catalyzed by cytochrome P450 enzymes and glycosyltransferases, which is critical for maintaining structural integrity and normal cellular function in the host. Introducing lengthy heterologous pathways consumes substantial cellular resources, including ATP, NAD(P)H, and precursor metabolites—imposing a metabolic burden that can compromise cell growth and decrease the yield of the target product.

#### 5.1.3. Scaling-Up Challenges

The scale-up of microbial synthesis of ginsenosides faces multiple challenges, including: heterogeneous distribution of dissolved oxygen, pH, and substrates in large-scale bioreactors, leading to cellular metabolic stress and fluctuating production yields; fluid shear stress generated by high-speed agitation and aeration, which can damage the fragile cell membranes of engineered microbial strains; the lack of real-time sensors for monitoring specific ginsenoside concentrations, resulting in delayed control feedback; foaming exacerbated by the surfactant properties of saponins, increasing the risk of microbial contamination; and the challenges associated with downstream separation and purification processes for the final product.

### 5.2. Engineered Biosynthesis of Ginsenosides: A Sustainable Platform

Synthetic biology platforms offer alternative pathways for ginsenoside production distinct from conventional agriculture, presenting advantages in environmental footprint, resource efficiency, and production capacity. This establishes the foundation for a more sustainable ginseng industry. Besides variable yields and a substantial environmental footprint, traditional field cultivation of *ginseng* requires extended growth cycles and intensive land use. In contrast, plant cell culture systems significantly reduce land and water usage, eliminate pesticide requirements, and maintain controlled environmental conditions [139].

The advancement of synthetic biology has revolutionized ginsenoside biosynthesis. The construction of engineered cell factories for heterologous ginsenoside production presents an effective strategy to enhance yields. Compared with traditional production methods, this approach offers notable advantages: reduced production cycles, utilization of cost-effective substrates, scalable and controllable fermentation processes, minimal spatial requirements, and consistent product quality independent of weather and environmental conditions. To enhance biosynthetic efficiency, several research priorities warrant investigation. First, thorough exploration and optimization of key genes and proteins within ginsenoside biosynthesis pathways are essential. This will establish fundamental genetic components for heterologous pathway assembly and enable efficient synthetic systems. Second, conducting protein engineering studies on enzymes is crucial. By directed evolution and rational design combined with high-throughput screening, catalytic efficiency and activity of essential biosynthetic enzymes can be improved, thereby increasing ginsenoside titers in chassis cells. Finally, developing and constructing chassis cells with enhanced traits will provide a solid foundation for establishing high-yield artificial biosynthesis platforms.

### 5.3. Integration of New Technologies with Plant-Based Chassis for Ginsenoside Biosynthesis

Plant-based chassis systems offer significant advantages for synthesizing plant secondary metabolites [140,141] (Figure 3D). Their inherent subcellular architecture, metabolic pathways, sophisticated enzymatic machinery, and regulatory transporters collectively facilitate precise biosynthesis of bioactive compounds while maintaining structural stability and reducing production and transportation costs. Plants sequester toxic intermediates using specialized organelles, preventing metabolic interference and enhancing product safety. Large-scale production can be achieved by either field cultivation or callus suspension cultures, enabling cost-effective biomass generation at commercial volumes. The rapid advancement of plant omics big data is expected to significantly enhance the utility of plant chassis in synthetic biology applications. Synthetic biology applications in rice have produced new germplasms, including Purple Endosperm Rice with anthocyanins, aSTARice with astaxanthin, and theaRice with theanine [142,143,144]. These examples demonstrate that plant-based chassis systems provide promising applications for terpenoid biosynthesis, with crops such as rice and sugarcane offering particularly strong advantages as synthetic biology platforms.

Microalgal cultivation enables high-density and rapid growth, along with amenable genetic traits. As predominantly photoautotrophic organisms, microalgae offer the potential for sustainable production, making them excellent hosts for synthesizing plant secondary metabolites successfully achieved the synthesis of dammarenediol-II at a titer of 2.6 mg/L by co-expressing PgDDS, a cytochrome P450 enzyme gene (PgCYP716A47), and a NADPH-cytochrome P450 reductase gene (AtCPR), under methyl jasmonate elicitation. This work lays a foundation for the production of ginsenosides in algal systems [145]. Plant chassis application for terpenoid biosynthesis remains restricted, primarily due to challenges including insufficient precursor supply, extended biosynthetic pathways requiring multiple genes, low catalytic activity of specialized enzymes and/or bioactive ginsenosides exhibiting low abundance, poor water solubility, and limited bioavailability in plant systems [146]. Successful biosynthesis of partial ginsenoside-related metabolites has been achieved in *Nicotiana tabacum* by genetic engineering. For instance, heterologous DS gene expression in engineered tobacco enabled successful DD biosynthesis, concurrently enhancing resistance against tobacco mosaic virus [41]. Co-overexpression of *DS*, *CYP716A47*, and *CYP716A53v2* in transgenic tobacco generated trace amounts of DD, PPD, and PPT, but concurrently induced male sterility [147]. Although microbial fermentation systems demonstrate significant advantages in yield potential and sustainability, their production and purification processes substantially affect the environment. The two biosynthesis modalities establish complementary dynamics: microbial systems excel at producing structurally simple, high-volume mainstream saponins, whereas plant platforms are superior for synthesizing complex, multi-step modified rare saponins. The future direction involves developing hybrid production platforms to synergistically leverage their respective strengths.

Furthermore, advanced gene editing technologies, particularly the sophisticated CRISPR/Cas systems, including the newly developed, highly efficient gene editing vectors (ScCas9^++^ and Cas12 variant) and plant high-efficiency dual base editors (e.g., PhieDBEs and TadDBE), provide powerful tools for precision genetic engineering [148,149,150,151,152,153]. Strategic knockout of genes in the glycolysis, tricarboxylic acid cycle, and glyoxylate shunt pathways enhances cytosolic Acetyl-CoA accumulation, thus improving precursor availability for ginsenoside biosynthesis [151,152,153]. Artificial chromosome and genome synthesis technologies are advancing, enabling the creation of a specialized “super-chassis” by de novo design and assembly of optimized genomes dedicated to saponin production. Building on this foundation, artificial intelligence (AI) and Large Language Models-driven optimization tools (e.g., PlantGPT) facilitate the establishment of efficient metabolic engineering frameworks [154]. Machine learning and deep learning technologies are transforming the field of metabolic engineering. By analyzing extensive omics datasets, AI systems can predict enzyme functions, design optimal metabolic pathways, and develop strategic solutions. For instance, the directed evolution of *UGT45* yielded high-efficiency mutants, and their chromosomal integration into the ZW04BY-RS strain enhanced RH2 production by 1.7-fold [94]. These technologies have been extensively implemented in microbial systems while remaining underutilized in plant contexts.

In conclusion, synthetic biology presents promising avenues to achieve efficient and sustainable production of ginsenosides. Through continued scientific research and technological innovation, combined with integrated assessment of economic viability, regulatory frameworks, and ethical implications, synthetic biology can transform ginsenoside supply paradigms, while significantly contributing to global health industries and agricultural sustainability.

## Figures and Tables

**Table 1:** Ginsenoside Varieties and Production Levels in Microbial Synthesis Systems.

Rare Ginsenoside	Synthetic Metabolic Engineering Strategies	Microbial Chassis	Yield	Reference
Ginsenoside CK	*tHMGR*, *PPD*, *ATR2-1*, *UGTPg1*	*S. cerevisiae*	1.4 mg/L	[30]
	*tHMG1*, *ERG9*, *ERG20*, *OpDDs*, *PPDDS*, *ATR1*, *UGT1*	*Yarrowia lipolytica*	161.8 mg/L	[86]
	*Synpggdds*, *SynPgPPDS*, *VvCPR*, *Pn3-29*, *PGM1*, *PGM2*, *UGP1*	*S. cerevisiae*	1.17 g/L	[87]
	*PGM2*, *UGP1*, *UGT1*	*S. cerevisiae*	1.7 g/L	[88]
	*tHMG1*, *PgCPR1*, *ERG1*, *ERG20*, *ERG9*, *PgDDS*, *PgPPDS*, *ERG12*, *ERG13*, *ERG8*, *ERG19*, *IDI*, *ERG10*, *PgUGT1*, *UGP1*, *PGM2*, *YNK1*, *ALG5*	*S. cerevisiae*	5.74 g/L	[89]
Ginsenoside Rg2	*CYP716A53v2*, *PgUGT71A54*, *PgURT94*, *RHM*	*S. cerevisiae*	1.3 g/L	[45]
Ginsenoside Rg3	*PgUGT74AE2*, *PgUGT94Q2*	*S. cerevisiae*	1.3 mg/L	[56]
	*PgDDS*, *PgPPDS*, *ATR2.1*, *tHMG1*, *ERG20*, *PgERG1*, *ERG9*, *PgUGT45*, *PgUGT29*	*S. cerevisiae*	3.49 μmol/g (DCW)	[90]
	*PnUGT33*	*S. cerevisiae*	51 mg/L	[91]
Ginsenoside F1	*ERG20*, *ERG1*, *ERG9*, *tHMG1*, *CYP716A53v2*, *PgCPR1*, *UGTPg100*	*S. cerevisiae*	42.1 mg/L	[60]
Ginsenoside F2	*PgDS*, *tHMG1*, *PgPPDS*, *IDI*, *AtATR2*, *PgUGT1*, *UGT74AE2*, *ERG20*, *ERG9*, *ERG1*, *ERG7*, *HAC1*, *PGM1*, *PGM2*, *UGP1*	*S. cerevisiae*	21.0 mg/L	[92]
Ginsenoside Rh1	*ERG20*, *PgERG1*, *ERG9*, *tHMG1*, *CYP716A53v2*, *PgCPR1*, *UGTPg100*	*S. cerevisiae*	98.2 mg/L	[60]
Ginsenoside Rh2	*ERG20*, *PgERG1*, *ERG9*, *tHMG1*, *M7-1*, *PGM.1*, *UGP1*, *PgPPDS*	*S. cerevisiae*	300 mg/L	[93]
	*PgDDS*, *synPgPPDS*, *ATR2.1*, *tHMG1*, *ERG20*, *ERG1*, *ERG9*, *UGT45*	*S. cerevisiae*	1.45 μmol/g (DCW)	[90]
	*ERG20*, *ERG9*, *ERG1*, *tHMG1*, *DS*, *CYP716A47-ATR1*, *RG12*, *ERG13*, *ERG19*, *ERG8*, *IDI*, *ERG1*, *tHMG1*, *UGTPn17*,	*S. cerevisiae*	354.69 mg/L	[61]
	*tHMG1*, *synPgCPR1*, *ERG1*, *ERG20*, *ERG9*, *synDD*, *SynPPDS*	*S. cerevisiae*	2.25 g/L	[94]
	*AtSuSy*	*E. coli*	0.20 g/L	[95]
	*AtSuSy*, *M315F*	*E. coli*	3.7 g/L	[96]
Ginsenoside Re	*CYP716A53v2*, *PgUGT71A53*, *PgUGT71A54*, *PgURT94*, *RHM*	*S. cerevisiae*	3.6 g/L	[12]
Ginsenoside *Ro*	*GgbAS*, *MtCPR*, *MtCYP716A12*, *tHMG1*, *ERG9*, *ERG1*	*S. cerevisiae*	528.0 ± 18.0 mg/L	[42]

Ginsenoside production. In Table 1, ATR: NADPH-cytochrome P450 reductase; *VvCPR*, Vitis vinifera Cytochrome P450 Reductase; *Pn3-29*, *Panax notoginseng* Glycosyltransferase 3-29; *PGM*, Phosphoglucomutase; *YNK*, Yeast Nucleoside Diphosphate Kinase; *ALG*, Asparagine-linked glycosylation; *RHM*, Rhamnose Synthase; *M7-1*, *Panax ginseng* Glycosyltransferase M7-1; *SuSy*, sucrose synthase; *M315F*, Mutant of *Panax ginseng* Protopanaxadiol Synthase.

**Table 2:** Pharmacological Activities and Underlying Mechanisms of Key Rare Ginsenosides.

Rare Ginsenosides	Pharmacological Activity	Effects	Function	Reference
Ginsenoside CK	Anticancer	Caspase-8 plays a key role in Compound K-stimulated apoptosis via the activation of caspase-3 directly or indirectly through Bid cleavage	Inhibit the viability of HL-60 cells, andan IC50 values of 14 muM	[108]
	Anti-diabetes	By elevating plasma adiponectin levels, hepatic glucose metabolism shifts from gluconeogenesis to glycogenesis, consequently improving insulin sensitivity and leading to upregulated expression of lipogenic genes and glucose transporters in adipose tissue	Oral glucose tolerance test (OGTT) using mice, revealed that CK improved glucose tolerance	[109]
	Anti-inflammatory	Inhibited prostaglandin E2, inducible NO synthase (iNOS) and COX-2 proteins expression in lipopolysaccharide (LPS)-induced RAW264.7 cell	Inhibited LPS-induced RAW 264.7 cells, IC50 values of 0.012 and 0.004 mM	[110]
	Anti-allergy	May ameliorate contact dermatitis or psoriasis by regulating COX-2 produced by macrophages cells and interferon-γ IL-5 and IL-4 induced by Th cells.	The level in interferon-γ IL-5 and IL-4 model group decreased 60–70%; 70–80%; 80–90%	[111]
	Anti-angiogenesis	It exerts anti-angiogenic activity by inhibiting p38 MAPK and AKT in HUVECs, with potential as a cancer chemopreventive agent.	Increased the phosphorylation level of p38(50%), blocked the AKT phosphorylation induced (10–20%).	[112]
	Antioxidant	Up-regulated the gene of hyaluronan synthase2 (HAS2), increased hyaluronan (HA) production in HaCaT cells.	The expression level in HaCaT cells was increased by 2.5-fold	[113]
	Anti-central neuroinflammatory disorders	Reduces the volume of ischemic cerebral infarction induced by middle cerebral artery occlusion and suppresses the activation of microglia in the ischemic cortex	The infarction volume was significantly reduced by 30–40%.	[114]
Ginsenoside Rh2	Anti-anxiety, anti-dementia	Can induce an increase in PACAP to activate PAC1, but not estrogen receptor, and thereby leads to attenuate Abeta-induced toxicity	The expression of PACAP and PCA1 mRNA increased by 200–250% and 180–220%.	[115]
	Anti-obesity	Can promote preadipocytes differentiation through activating glucocorticoid receptor (GR).	The fat-producing capacity of 3T3-L1 cells was increased by 2.2 times, and the expression of adipogenic genes was upregulated by 2 to 8 times.	[116]
		Activation of the AMPK signaling pathway leads to the phosphorylation and inhibition of its downstream target ACC, ultimately downregulating the expression of key adipogenic transcription factors PPARγ and C/EBPα.	The level of p-AMPKα and p-ACC increased by 2.0–2.5 times and 2.0–3.0 times.	[117]
	Anti-inflammatory	Significantly suppressed NF-kappaB and MAP kinase activities, which are upstream signaling molecules in inflammation.	Inhibit the degradation of IκB-α (by 70–80%) and the phosphorylation of ERK and p38 (by 60–80%)	[118]
	Anti-tumor	Rh2-mediated cell cycle arrest in human breast cancer cells is caused by p15 (Ink4B) and p27 (Kip1)-dependent inhibition of kinase activities of G(1)-S specific Cdks/cyclin complexes.	The expression of p27 (Kip1) protein and p15 (Ink4B) increased by 3 to 4 times and 2 to 3 times	[119]
		C_20_ may play an important role in antitumor activities.	IC50 values of 14 μM, induce 35% of cell apoptosis.	[120]
Ginsenoside F1	Antioxidant	By inhibiting p38 MAPK-dependent NF-κB activity, it suppresses the senescence-associated secretory phenotype (SASP) in D-galactose-induced astrocytes.	The survival rate of neurons has recovered to approximately 85–90%.	[121]
	Anti-atherosclerosis	Significantly increased A20 expression level and A20 siRNA markedly abolished the attenuation of GF1 on NF-κB nuclear translocation and inflammatory factors expression	The expression of A20 increased by 3.5 times, inhibiting approximately 70% of NF-κB pathway activations.	[122]
Ginsenoside Rg3	Anticancer	Can inhibit expression of HIF-1α and VEGF in human gastric cancer cells and may influence abdominal implantation metastasis of gastric cancer through inhibiting its expression.	Accumulation of HIF-1α protein and VEGF protein secretion lead to an inhibition rate of 60–70%	[123]
		Effectively suppressed the migration and invasion of liver cancer cells by upregulating the protein expression of ARHGAP9.	Inhibits the migration and invasion abilities of liver cancer cells by 70–75%, and increases the expression of ARHGAP9 by 3 times.	[124]
	Anti-osteoporosis	By activating the Nrf2/HO-1 signaling pathway, it enhances the body’s antioxidant capacity and alleviates oxidative stress-induced damage to bone cells. And, through activation of the Wnt/β-catenin signaling pathway, it upregulates osteogenesis- related factors (e.g., Runx2) and modulates the OPG/RANKL ratio, thereby promoting bone formation and inhibiting bone resorption.	The expression of β-catenin and Nrf2 protein in bone tissue increased by ~60–80% and 150–200%.	[125]
Ginsenoside Rg5	Anticancer	Decreased the phosphorylation levels of PI3K, Akt, mTOR, and Bad and suppressed the PI3K/Akt signaling pathway in breast cancer.	The IC50 values of 27–33 μM, induces approximately 40% cell apoptosis and inhibits approximately 50–60% of cell migration and invasion.	[126]
		May have a tumor-suppressive effect on esophageal cancer by promoting apoptosis and may be associated with the downregulation of the PI3K/Akt signaling pathway.	The expression of p-PI3K p85 and p-Akt (Ser473) protein was inhibited by ~50–60% and 60–70%.	[127]
	neuroprotective	Exerts its therapeutic effects mainly through PI3K/AKT, MAPK signaling pathways, and the regulation of apoptosis and cell cycle.	Protect neurons from damage (increase cell vitality by 30%).	[128]
Ginsenoside Rh4	Anti-anemia	Some positive regulators (EPO, erythroid transcription factor-1, and interleukin-3) related to hematopoiesis increased and some negative regulators (interferon-γ and tumor necrosis factor-α) decreased in vivo	The expression of Nrf2/HO-1 protein increased by 150–200%, inhibiting the apoptosis of bone marrow hematopoietic cells.	[129]
	Anti-tumor	Decreasing Bcl-2, increasing Bax, and activating caspase-8, -3 and PARP.	Repressing 60% of tumor growth	[130]
Ginsenoside Rs6	Anti-inflammatory	By suppressing the activation of p-STAT-1 (phosphorylated signal transducer and activator of transcription 1) and NF-KB (nuclear factor kappa- light-chain-enhancer of activated B cells), it blocks the inflammatory signaling pathway.	Inhibit the NF-κB and STAT1 pathway by 50–60%.	[131]
Ginsenoside Rk1	Anti-inflammatory	Inhibited the lipopolysaccharide-stimulated phosphorylation of NF-κB and janus kinase (Jak)2 and signal transducer and activator of transcription (Stat)3 at Ser727 and Tyr705.	The inhibition rates of p-Jak2 and p-Stat3 reached 80% and 90%.	[132]
	Antioxidation	Due to its antioxidation, antiapoptosis, anti- inflammation, and antinitrative effects in APAP- induced hepatotoxicity.	The apoptotic index of liver tissue (TUNEL) decreased by 70–80%	[133]
	Anticancer	Reduced the high expression of PD-L1 in lung adenocarcinoma cells by inhibiting NF-κB signaling.	By inhibiting the NF-κB pathway (80% for p-p65), 50% of tumor growth can be suppressed.	[134]
Ginsenoside Rk3	Anticancer	Inhibit Eca109 and KYSE150 cell proliferation through activating apoptosis and autophagy by blocking the PI3K/Akt/mTOR pathway.	The volume and weight of the tumor in the body was reduced by 50–60%, and 55–65%.	[135]
	Anti-inflammatory	Can improve DSS-induced ulcerative colitis by protecting intestinal barrier function and inhibiting NLRP3 inflammasome expression,	The length of the colon was improved by approximately 42%, tissue damage was reduced by about 60%,	[136]

## Data Availability

No new data were created or analyzed in this study.

## Abbreviations

The following abbreviations are used in this manuscript:

ginseng	*Panax ginseng*
MVA	Mevalonate
MEP	Methylerythritol phosphate
IPP	Isopentenyl diphosphate
DMAPP	Isomer dimethylallyl diphosphate
HMGR	HMG-CoA reductase
DXP	1-Deoxy-D-xylulose-5-phosphate
GPP	Geranyl diphosphate
DMAPP	3,3-Dimethylallyl diphosphate
FPP	Farnesyl pyrophosphate
FPS	Farnesyl pyrophosphate synthase
SQS	Squalene synthase
DDS	Dammarenediol synthase
CYP716A47	Cytochrome P450 oxidases enzymes
PPD	Protopanaxadiol
CYP716A53v2	Cytochrome P450 oxidases enzymes
PPT	Protopanaxatriol
β-AS	β-Amyrin synthase
CYP716A52v2	Cytochrome P450 oxidases enzymes
OA	Oleanolic acid
SQE	Squalene epoxidase
OSCs	Oxidosqualene cyclases
DD	Dammarenediol-II
DS	Dammarenediol-II synthase
CYP450	Cytochrome P450
UGTs	UDP-glycosyltransferases
*notoginseng*	*Panax notoginseng*
*E. coli*	*Escherichia coli*
*B. subtilis*	*Bacillus subtilis*
MeJA	Methyl jasmonic acid
JA	Jasmonic acid
CAGR	Compound annual growth rate
